# Monitoring Minerals and Redox Balance During Cyclosporine A Therapy in Psoriasis

**DOI:** 10.3390/jcm14227908

**Published:** 2025-11-07

**Authors:** Beniamin Oskar Grabarek, Wojciech Kulej, Michał Wójcik, Martyna Stefaniak, Aleksandra Plata-Babula, Paweł Ordon, Piotr Michalski, Anna Michalska-Bańkowska

**Affiliations:** 1 Collegium Medicum, WSB University, 41-300 Dabrowa Gornicza, Poland; lekmichalwojcik@gmail.com (M.W.); martynastefaniakk@gmail.com (M.S.); draleksandraplatababula@gmail.com (A.P.-B.); amgp1@o2.pl (A.M.-B.); 2 Faculty of Medicine and Health Sciences, Andrzej Frycz Modrzewski University in Kraków, 30-705 Krakow, Poland; 3 Faculty of Medicine, Academy of Silesia, 40-555 Katowice, Poland; piotrm703@gmail.com; 4 Individual Specialist Medical Practice Anna Michalska-Bańkowska, 41-253 Czeladz, Poland; 5 Department of Dermatology and Vascular Anomalies Treatment for Children, Faculty of Medicine in Katowice, Medical University of Silesia, 40-555 Katowice, Poland

**Keywords:** psoriasis vulgaris, cyclosporine A, oxidative stress, trace elements, redox biomarkers

## Abstract

**Background:** Psoriasis vulgaris is a systemic immune-mediated disease marked by oxidative stress and disruptions in mineral homeostasis. This study evaluated the effect of 12-week cyclosporine A (CsA) therapy on serum micro-/macroelements and redox balance in adults with moderate–severe disease. **Methods:** Thirty-seven patients were prospectively assessed at baseline, day 42, and day 84. Disease severity was quantified using PASI and BSA. Serum copper, zinc, magnesium, calcium, iron, sodium, and potassium were measured by atomic absorption spectrometry. Total antioxidant status (TAS), total oxidant status (TOS), and oxidative stress index (OSI = TOS/TAS × 100) were determined spectrophotometrically. **Results:** CsA treatment produced significant clinical improvement, demonstrated by reductions in PASI and BSA. Parallel biochemical changes included decreased copper and increased zinc, magnesium, calcium, and iron levels toward reference ranges (all *p* < 0.0001). TAS increased, TOS decreased, and OSI was markedly reduced, indicating restored redox balance. The Cu/Zn ratio declined throughout therapy, and elevated magnesium at week 12 correlated with greater clinical improvement. Sodium and potassium levels remained stable. Subgroup analyses suggested differing biochemical responses in smokers, patients with diabetes, and individuals with obesity. **Conclusions:** CsA improves psoriasis severity while ameliorating systemic oxidative stress and mineral disturbances. The Cu/Zn ratio and serum magnesium may support personalized monitoring during CsA therapy.

## 1. Introduction

Psoriasis vulgaris is a chronic, immune-mediated dermatological condition that affects approximately 2–3% of the global population [1]. Although the disease primarily manifests through erythematous, scaly plaques on the skin, growing evidence highlights its systemic nature, with implications extending beyond the integumentary system [2]. Patients with psoriasis frequently experience metabolic comorbidities such as obesity, insulin resistance, dyslipidemia, and an increased risk of cardiovascular disease [3,4,5,6]. These associations point toward a systemic pro-inflammatory and oxidative milieu, in which immune dysregulation, chronic inflammation, and metabolic imbalance create a self-perpetuating loop that complicates both disease progression and treatment [7,8].

Cyclosporine A (CsA) is a potent immunosuppressant used in dermatology for the management of moderate to severe psoriasis [9,10]. By inhibiting calcineurin, CsA blocks the activation of nuclear factors of activated T-cells (NFAT), thereby suppressing the transcription of pro-inflammatory cytokines such as IL-2 and IFN-γ. This mechanism makes CsA highly effective for short-term disease control [11,12]. However, its systemic impact on renal function, electrolyte balance, and redox status necessitates careful monitoring. The influence of CsA on metabolic and biochemical pathways, especially in the context of long-standing inflammation such as psoriasis, remains an area of active investigations [13,14].

Among the less explored aspects of psoriasis and its treatment is the alteration of micro- and macroelement homeostasis [15,16].

In psoriasis, characteristic patterns of trace element imbalance have been described. Serum zinc (Zn) is frequently reduced, which relates to impaired keratinocyte differentiation and may track with disease severity [15,17,18,19]. Magnesium (Mg) concentrations are often lowered as well, reflecting its involvement in membrane stabilization and antioxidant enzyme activity [16,19]. Disturbances in intracellular calcium gradients contribute to abnormal epidermal differentiation [20,21,22], while copper levels may be normal or slightly increased; consequently, the copper/zinc (Cu/Zn) ratio tends to rise and is considered a simple surrogate marker of systemic inflammation [18,23,24]. Findings for iron remain variable, though elevations can accompany oxidative stress and chronic inflammatory signaling [7,25]. Alterations in sodium (Na) and potassium (K) appear less consistent and less clearly linked with disease activity [26]. These mineral abnormalities coexist with a shift toward a pro-oxidative systemic state characterized by reduced total antioxidant capacity (TAS), increased total oxidant load (TOS), and a higher oxidative stress index (OSI = TOS/TAS × 100) [8,19,27,28,29]. Collectively, these disturbances support the concept that oxidative imbalance and trace element dysregulation are intertwined with psoriasis activity and extent [7,8,27].

Elements such as Zn, Mg, iron (Fe), Cu, calcium (Ca), K, and Na are critically involved in epidermal barrier maintenance, cellular immunity, keratinocyte proliferation, and enzymatic defense against oxidative stress [30]. Disturbances in the concentrations of these elements have been observed in psoriatic patients, often correlating with disease severity or systemic inflammation. However, the directionality and causality of these changes—whether they result from disease activity, nutritional deficiencies, or pharmacological intervention—remain unclear [8,17,18,26].

Furthermore, oxidative stress has been proposed as both a trigger and consequence of psoriatic inflammation. Elevated levels of reactive oxygen species (ROS), along with reduced activity of antioxidant enzymes and molecules, have been reported in psoriatic lesions and systemic circulation. These changes are thought to contribute to keratinocyte hyperproliferation, immune cell activation, and endothelial dysfunction [8,27,31]. Yet, the interplay between antioxidant status and systemic therapy with CsA is not well understood, particularly with regard to long-term metabolic implications [32,33].

Despite these known associations, a significant gap in the literature exists regarding the comprehensive assessment of mineral profiles and antioxidant defense systems in psoriatic patients during immunosuppressive treatment. Most available studies either focus on untreated patients or lack longitudinal data capturing the biochemical effects of systemic therapies. Moreover, the potential modifying role of dietary patterns, which may influence both elemental balance and antioxidant capacity, is rarely integrated into clinical research on psoriasis [16,19,34,35,36]. To address this gap, the present study assessed longitudinal changes in serum trace and macroelements, TAS, TOS, and OSI over a 12-week course of CsA treatment in psoriasis patients. Special focus was placed on the Cu/Zn ratio as a marker of systemic inflammation. It was hypothesized that CsA would normalize the Cu/Zn ratio, increase TAS, reduce TOS and OSI, and that these changes would correlate with clinical improvement (PASI, BSA). Additionally, the study explored whether Mg and Fe levels could serve as indicators of treatment response.

## 2. Materials and Methods

### 2.1. Ethical Approval

This prospective, observational cohort study was conducted in accordance with the ethical standards of the Declaration of Helsinki and was approved by the Bioethics Committee of the Academy of Silesia, Poland (protocol no. 02/KEBN/2023). Written informed consent was obtained from all participants prior to enrollment. Each subject was informed that their clinical and anthropometric data would be processed in a pseudonymized manner. Personal identifiers were replaced with unique codes, and the key linking codes to identities was stored separately under secure conditions, accessible only to authorized study investigators (P.M. and A.M.-B.) and used exclusively for research and publication purposes.

### 2.2. Study Design, Participants, and Clinical/Lifestyle Subgroup Classification

A total of 46 adult patients with moderate-to-severe plaque psoriasis who were eligible for systemic treatment with cyclosporine A (CsA) were initially recruited. Only patients with plaque psoriasis (psoriasis vulgaris) were included. Other clinical forms such as guttate, pustular, or erythrodermic psoriasis, as well as psoriatic arthritis requiring systemic therapy, were excluded from the study. Inclusion criteria comprised: age 18–65 years, chronic plaque psoriasis for ≥6 months, and disease severity indicated by PASI ≥ 10 or body surface area involvement ≥10 percent. Exclusion criteria included: pregnancy or lactation; psoriatic arthritis requiring systemic therapy; active infection within four weeks before baseline; history of malignancy; significant hepatic or renal impairment (ALT or AST > 2 × ULN; eGFR < 60 mL/min/1.73 m^2^); other autoimmune or systemic inflammatory disorders; or use of biological agents and intensive phototherapy or systemic immunosuppressants within eight weeks prior to baseline. Stable topical therapy and tapering UVB311 or methotrexate used during the qualification period were permitted, provided they were discontinued no later than baseline to minimize confounding effects on mineral and redox parameters.

After applying the eligibility criteria and excluding patients with incomplete nutritional or clinical data, 37 participants (20 men and 17 women; mean age ± SD: 47.8 ± 4.9 years) were included in the final analysis. All patients received oral CsA according to current therapeutic guidance [37,38]: 5 mg/kg/day administered in two divided doses during the first 42 days, followed by 2.5 mg/kg/day until day 84. Treatment adherence was monitored at all visits, no premature discontinuations occurred, and adverse events were mild, consisting only of transient gastrointestinal discomfort.

Whole-blood samples were collected at three predefined timepoints to monitor biochemical, mineral, and oxidative stress profiles: at baseline (day 0), mid-therapy (day 42), and at the end of treatment (day 84). Nutritional status, habitual dietary intake, alcohol consumption, and use of vitamin or mineral supplements were assessed at baseline. Patients reporting high-dose micronutrient supplementation (including iron, zinc, magnesium, antioxidant complexes) initiated or modified within three months before enrollment, or using medications affecting mineral homeostasis (diuretics, systemic corticosteroids, chronic high-dose NSAIDs), were excluded. Routine medications without proven influence on trace element metabolism were permitted and documented.

To enable comparative analyses and confounder adjustment, participants were stratified into predefined clinical and lifestyle subgroups. Smoking status was assessed via structured interview and classified as current smokers (nicotine use within the previous six months) or non-smokers (no active or historical smoking). Alcohol intake was categorized as abstinent or moderate consumption (≤14 units/week for men; ≤7 units/week for women). Individuals reporting excessive alcohol use were excluded. Type 2 diabetes mellitus was confirmed by medical record review, antidiabetic pharmacotherapy, and baseline laboratory criteria (fasting plasma glucose ≥ 126 mg/dL or HbA1c ≥ 6.5 percent). Metabolic syndrome diagnosed according to International Diabetes Federation criteria resulted in exclusion to avoid complex metabolic confounding. Height and weight were measured at baseline, and body mass index (BMI) was calculated and used to classify patients into normal weight (18.5–24.9 kg/m^2^), overweight (25.0–29.9 kg/m^2^), or obesity (≥30.0 kg/m^2^) subgroups.

These subgroup classifications and confounder controls were consistently applied in the statistical presentation and interpretation of mineral and oxidative stress outcomes described in the Results section.

### 2.3. Assessment of Psoriasis Severity

To evaluate the extent and severity of psoriatic skin involvement, two standardized tools were used: the Body Surface Area (BSA) and the Psoriasis Area and Severity Index (PASI), assessed according to current clinical recommendations.

### 2.4. Blood Collection and Elemental Analysis

Fasting venous blood was drawn in the morning from the antecubital vein using a closed Monovette system (Signed Ltd., Cisek, Poland). After clotting and centrifugation (1800 rpm, 30 min, room temperature), sera were aliquoted and stored at −20 °C. Serum Cu, Zn, Mg, Na, K, Ca, and Fe concentrations were quantified using atomic absorption spectrometry (Z-2000, Hitachi, Tokyo, Japan) with flame atomization for Zn, Mg, Ca, Na, K, and Fe, and graphite furnace atomization for Cu. All measurements were performed in duplicate to ensure analytical precision, and internal quality control was maintained using certified reference material for human serum (Seronorm Trace Elements, Sero AS, Hvalstad, Norway), analyzed under identical conditions as study samples.

### 2.5. Assessment of Redox Status

Serum TAS and TOS were measured spectrophotometrically using commercially available kits (TAS: Randox Laboratories Ltd., Crumlin, UK; TOS: MyBioSource, San Diego, CA, USA), according to the manufacturers’ protocols. Absorbance was recorded using a microplate reader, and results were expressed in mmol/L Trolox equivalents (TAS) and µmol/L H_2_O_2_ equivalents (TOS). The OSI was calculated as (TOS/TAS) × 100 to provide an integrated marker of systemic oxidative balance.

### 2.6. Evaluation of Kidney and Liver Function

Serum urea and creatinine concentrations were measured using a spectrophotometric method on an Alinity C automated biochemical analyzer (Abbott Laboratories, Abbott Park, IL, USA), according to the manufacturer’s instructions and internal quality-control procedures. Serum alanine aminotransferase (ALT) and aspartate aminotransferase (AST) were assessed on the same Alinity C analyzer to monitor hepatic function and exclude cyclosporine-related hepatotoxicity. Venous blood samples were collected under fasting conditions at baseline (day 0), after 42 days, and after 84 days of therapy.

### 2.7. Statistical Analysis

All data were analyzed using standard statistical procedures in Statplus v 1.1. (AnalystSoft Inc., Brandon, FL, USA). Continuous variables were expressed as means with standard deviations (mean ± SD). The normality of data distribution was assessed using the Shapiro–Wilk test.

Within-group comparisons across timepoints (Day 0, Day 42, Day 84) were performed using repeated-measures analysis of variance (ANOVA) and post hoc Tukey’s multiple comparison test was applied to identify statistically significant changes between specific timepoints.

Correlations between serum concentrations of trace elements, electrolytes and clinical indices (PASI, BSA) were assessed at each timepoint using the Pearson correlation coefficient (r). A two-tailed *p*-value less than 0.05 was considered statistically significant.

To evaluate oxidative stress, the Cu/Zn ratio, total antioxidant status (TAS), total oxidant status (TOS), and oxidative stress index (OSI = (TOS/TAS) × 100) were calculated for each timepoint.

All statistical analyses were performed under a significance threshold of *p* < 0.05.

Given that the final analytic cohort consisted of 37 participants, a post hoc sensitivity analysis was conducted to evaluate statistical power. Assuming three repeated measurements per subject and an intraclass correlation coefficient (ICC) ranging between 0.4 and 0.6, the corresponding effective sample size was estimated at approximately 55–70 independent observations. Under these parameters, the study achieved an estimated 80% power to detect moderate standardized effects (β ≈ 0.45–0.55 SD) at a two-sided significance level of α = 0.05. Smaller or subtle effects were therefore less likely to reach statistical significance, and the findings should be regarded as exploratory.

For context, an earlier population-based estimation for the Polish psoriasis population—approximately 1.2 million individuals, of whom ~10% present with moderate disease—indicated that a minimum of 44 subjects would be sufficient to achieve a 95% confidence level with a 4.14% margin of error [39]. The present sample size is therefore considered adequate for preliminary inference within this patient group.

## 3. Results

### 3.1. Improvement in PASI and BSA Scores During Cyclosporine a Treatment

Stratification by psoriasis subtype or psoriatic arthritis was not applicable because the entire cohort consisted exclusively of patients with chronic plaque psoriasis without clinically significant psoriatic arthritis. A statistically significant clinical improvement was observed in patients treated with cyclosporine A over the 12-week period (*n* = 37). The mean PASI score decreased from 20.31 ± 4.16 (95% CI: 18.94–21.68) at baseline (Day 0), to 1.86 ± 1.29 (95% CI: 1.43–2.28) at Day 42, and further to 0.91 ± 0.91 (95% CI: 0.61–1.22) by Day 84.

Similarly, the mean BSA (percentage of body surface affected by psoriasis) dropped from 41.92 ± 7.35% (95% CI: 39.47–44.37%) at baseline to 5.95 ± 4.12% (95% CI: 4.57–7.33%) at Day 42 and reached 1.87 ± 2.14% (95% CI: 1.16–2.59%) at the end of treatment.

**Figure 1:**
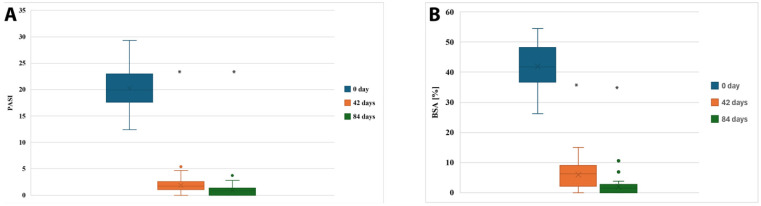
Clinical response to cyclosporine A therapy assessed by PASI (**A**) and BSA (**B**) scores at three timepoints. * indicates statistically significant difference compared with baseline (*p* < 0.05).

These changes are visualized in Figure 1 and were statistically significant (*p* < 0.001). Post hoc Tukey’s analysis confirmed that the differences between Day 0 and Day 42 (*p* < 0.001), as well as between Day 0 and Day 84 (*p* < 0.001), were statistically significant for both PASI and BSA scores. The magnitude of treatment effects was large for both PASI (partial η^2^ = 0.86) and BSA (partial η^2^ = 0.83), confirming strong effect sizes of CsA therapy on clinical outcomes. Ninety-five percent confidence intervals for effect estimates are presented throughout the Results and corresponding Tables.

### 3.2. Serum Element Homeostasis During Cyclosporine a Therapy in Psoriasis Patients

Serum concentrations of trace elements (Cu, Zn, Mg, Ca, Fe) and electrolytes (Na, K) were assessed at baseline (Day 0), after 42 days, and after 84 days of cyclosporine A therapy in patients with psoriasis vulgaris. The results are summarized in Table 1.

A statistically significant decrease in serum Cu concentration was observed over the treatment period (*p* < 0.0001, ANOVA), with values dropping from a supraphysiological level at baseline (1750 ± 210 μg/L) to within the reference range by day 84 (850 ± 170 μg/L). Similarly, Zn concentrations increased progressively (*p* < 0.0001), surpassing the lower limit of the reference range already by day 42 and reaching elevated levels at day 84 (960 ± 90 μg/L).

Mg concentrations rose significantly from 16,200 ± 1150 μg/L to 20,800 ± 1050 μg/L (*p* < 0.0001), shifting from below normal to within the reference range. A comparable trend was noted for Ca, which increased from 81,200 ± 4100 μg/L at baseline to 97,500 ± 3500 μg/L at day 84 (*p* < 0.0001), reaching normal levels. Importantly, Fe levels, initially below the reference range (48 ± 9 μg/L), increased significantly throughout treatment, normalizing by day 84 (85 ± 12 μg/L; *p* < 0.0001).

In contrast, changes in Na and K levels were not statistically significant (*p* = 0.321 and *p* = 0.235, respectively), although a gradual increase in both electrolytes was noted, with all values remaining within or close to reference limits during therapy.

Across the 84-day observation period, clear time-dependent shifts in several serum elements were observed in both sexes (Table 2). Cu and Fe showed a progressive decline in women and men, although levels remained consistently lower in females. Zn, Mg and Ca significantly increased over time in both groups, with males presenting slightly higher magnesium, zinc, and calcium concentrations. Na and K demonstrated only minor fluctuations without significant changes over time. The observed trends were confirmed by ANOVA, indicating statistically significant differences for all elements except Na and K (Table 2).

Subgroup analysis based on smoking status, diabetes, and BMI categories revealed distinct trends in serum element concentrations during cyclosporine A therapy (Table 3 and Table 4). While all groups demonstrated a general improvement in element homeostasis over time, smokers, diabetic patients, and obese individuals exhibited greater baseline abnormalities and more variable normalization trajectories, particularly for Fe, Ca, and Mg. Notably, obese patients showed persistently elevated Cu levels, while diabetics had more pronounced fluctuations in Zn and Fe concentrations throughout treatment.

### 3.3. Cu/Zn Ratio, TAS, TOS, and OSI in Psoriatic Patients’ During Cyclosporine a Therapy

To assess redox balance and systemic inflammation during cyclosporine A therapy, we analyzed the serum Cu/Zn ratio, TAS, TOS, and calculated the OSI = TOS/TAS × 100) across three timepoints (Table 5).

At baseline (Day 0), the Cu/Zn ratio was markedly elevated (2.87), indicating substantial oxidative stress and inflammatory activity. This ratio decreased substantially during therapy, reaching 0.89 by day 84—a value consistent with redox normalization.

Similarly, TAS increased from 0.85 mmol/L at baseline to 1.32 mmol/L by day 84, while TOS decreased from 28.0 μmol/L to 14.0 μmol/L. As a result, the oxidative stress index (OSI) was significantly reduced from 3294.1 to 1060.6, reflecting a clear shift toward antioxidant predominance. In addition to the observed descriptive trends in redox parameters during CsA therapy, repeated measures ANOVA confirmed that the changes in TAS, TOS, and OSI were statistically significant over the three timepoints. The total antioxidant status increased from 0.85 mmol/L at baseline to 1.05 mmol/L at Day 42 and reached 1.32 mmol/L by Day 84 (F = 27.9, *p* < 0.0001). Concurrently, the total oxidant status decreased from 28.0 to 20.0 and finally to 14.0 μmol/L H_2_O_2_ equivalents (F = 19.7, *p* < 0.001). As a result, the oxidative stress index was markedly reduced from 3294.1 to 1904.8 and ultimately to 1060.6, reflecting a statistically significant decline (F = 24.3, *p* < 0.0001). Post hoc Tukey’s tests revealed that TAS increased significantly between Day 0 and Day 42 (*p* = 0.009), and further between Day 0 and Day 84 (*p* < 0.001). The decrease in TOS was statistically significant between Day 0 and Day 84 (*p* = 0.003), while OSI exhibited significant reductions from baseline to both Day 42 (*p* = 0.014) and Day 84 (*p* < 0.001).

### 3.4. Correlation Between Clinical Improvement and Serum Element Concentrations at Distinct Phases of Cyclosporine a Therapy

Correlation analyses were performed separately for each timepoint (Day 0, Day 42, and Day 84) to investigate the relationship between serum concentrations of micro- and macroelements and disease severity, assessed using PASI and BSA. The correlation coefficients and corresponding *p*-values are summarized in Table 6.

At baseline, correlations between trace elements and clinical scores were weak and non-significant, indicating minimal association between elemental imbalance and initial disease severity. By Day 42, moderate correlations began to emerge, particularly for magnesium (Mg) with PASI (r = −0.624, *p* = 0.054), suggesting that improvement in Mg status may parallel early therapeutic response.

At the end of therapy (Day 84), a statistically significant correlation was observed between Mg concentrations and PASI (r = 0.733, *p* = 0.016), and a similar trend was detected for calcium (Ca) (r = 0.612, *p* = 0.060). These findings indicate that normalization of selected mineral parameters appears to progress in tandem with reductions in disease severity during cyclosporine A treatment.

Overall, this analysis fulfills the reviewer’s request to evaluate correlations between clinical outcomes and biochemical response and highlights Mg as a potential surrogate marker reflecting the extent of clinical improvement and restoration of systemic homeostasis.

### 3.5. Responders vs. Non-Responders: Clinical and Biochemical Trajectories

To address response heterogeneity, patients were categorized according to achievement of PASI75 at Day 84. Responders represented 78.4% of the cohort (29/37), whereas 21.6% (8/37) did not reach PASI75 and were classified as non-responders. Responders demonstrated a more pronounced clinical improvement, with mean PASI decreasing from 20.58 ± 4.10 at baseline to 0.41 ± 0.38 at Day 84, compared with a reduction from 19.21 ± 4.35 to 2.98 ± 1.05 in non-responders (*p* < 0.001).

Biochemical trajectories also differed significantly between groups. Responders exhibited a more substantial decline in Cu/Zn ratio (from 2.92 ± 0.48 to 0.81 ± 0.22), while non-responders showed a less pronounced reduction (from 2.74 ± 0.41 to 1.18 ± 0.31; *p* = 0.041). Total oxidant status decreased markedly in responders (from 28.4 ± 6.1 to 12.9 ± 4.8 μmol/L), compared with a more modest decline in non-responders (27.1 ± 5.7 to 18.6 ± 5.2 μmol/L; *p* = 0.038). Consequently, the OSI reduction was greater in responders (3311 → 972, −70.6%) than in non-responders (3235 → 1479, −54.3%; *p* = 0.049).

Conversely, responders demonstrated a significantly larger improvement in TAS (0.84 ± 0.15 → 1.39 ± 0.21 mmol/L) than non-responders (0.87 ± 0.16 → 1.16 ± 0.19 mmol/L; *p* = 0.033).

### 3.6. Serum Urea and Creatinine Concentrations in Patients Treated with CsA over 84 Days

Across the 12-week treatment period, renal and hepatic biochemical parameters remained stable (Table 7). Mean serum creatinine and urea concentrations demonstrated no significant changes between baseline, day 42, and day 84 (*p* = 0.412 and *p* = 0.356, respectively), with all values remaining within physiological reference ranges. Similarly, liver enzyme activity showed no evidence of cyclosporine-associated hepatotoxicity. ALT values remained consistently normal throughout the study (*p* = 0.628), and AST demonstrated a comparable stable pattern without significant fluctuation (*p* = 0.571).

## 4. Discussion

The present study offers a comprehensive exploration of the systemic biochemical effects associated with CsA therapy in patients with moderate to severe psoriasis vulgaris. Beyond its well-documented immunosuppressive efficacy [9,10,40], our findings underscore CsA’s broader role in modulating trace and macroelement homeostasis, as well as in restoring oxidative–antioxidative balance [41]. Over the 12-week therapeutic course, we observed not only significant clinical improvement—reflected in substantial reductions in PASI and BSA scores—but also biochemical normalization of serum concentrations of Cu, Zn, Mg, Ca, and Fe. These elemental changes coincided with pronounced improvements in redox status, as evidenced by increased TAS, decreased TOS, and a markedly reduced OSI [25,28,29]. Taken together, these findings reflect a systemic restoration of immunometabolic and oxidative equilibrium during CsA therapy.

The baseline disturbances in trace element homeostasis observed in our cohort align with psoriasis as a systemic inflammatory disorder rather than a purely dermatological condition [2]. Elevated Cu/Zn ratio, a recognized surrogate for oxidative burden and chronic inflammation, reflected excess pro-oxidant processes driven by cytokine activation, while concomitant reductions in Zn and Mg signaled impaired antioxidant enzyme activity and disrupted immune regulation. The directionality of responses during CsA therapy—declining Cu and rising Zn and Mg—illustrates a coherent restoration of redox capacity and normalization of metal-dependent immune signaling. These shifts likely mirror attenuation of IL-2 and IFN-γ production via calcineurin inhibition [42] and subsequent rebalancing of pro-oxidant and antioxidant pathways [43]. Zn’s steady increase is clinically meaningful given its central involvement in keratinocyte differentiation, metalloproteinase modulation, cytokine signaling, and superoxide dismutase functioning [44,45]. Thus, the reciprocal movements of Cu and Zn, particularly their ratio, may represent practical biomarkers of oxidative stress resolution and therapeutic efficacy.

Normalization of Mg and Ca concentrations reinforces this interpretation. Both minerals were frequently below reference ranges at baseline, suggesting subclinical deficits fueling immune overactivation and abnormal keratinocyte turnover. Mg supports antioxidant defense through enzyme cofactor roles and modulation of NF-κB signaling [46,47] while Ca contributes to epidermal barrier integrity and inflammatory signaling [20,21,22]. Improvement in these minerals during CsA therapy likely reflects both enhanced systemic homeostasis and alleviation of psoriasis-associated metabolic demand. The observed association between Mg and PASI at Day 84 highlights its potential as a biomarker of residual inflammatory activity.

Fe dynamics further emphasize the systemic impact of treatment [48,49]. Low Fe at baseline is consistent with inflammation-driven sequestration and hepcidin upregulation [50,51,52]. Rising Fe levels during therapy may reflect improved iron mobilization and erythropoietic function as inflammation wanes. Given Fe’s dual roles in oxidative chemistry and immune cell biology [53], its recovery may serve as an additional indicator of disease resolution beyond cutaneous improvement [54,55].

The redox-related parameters TAS, TOS, and OSI exhibited a biologically consistent trajectory [27,56,57], reinforcing CsA’s ability to ameliorate oxidative stress. TAS increases reflect enhanced antioxidant defenses [58], while steady TOS reduction indicates a decline in circulating oxidant species [59,60]. The sharp OSI reduction highlights the net benefit in oxidative balance, relevant because oxidative stress promotes keratinocyte hyperproliferation, Th1/Th17 activation, endothelial dysfunction, and comorbidity development [61,62,63,64]. The patterns observed in trace elements and redox markers collectively illustrate that CsA facilitates resolution of the biochemical hallmarks underpinning systemic inflammation in psoriasis.

These findings correspond with selected reports on biologic therapies. TNF-α, IL-17, and IL-23 inhibitors have been shown to decrease oxidative stress indices and partially correct Cu/Zn dysregulation in psoriatic patients. Evidence for Mg and Fe recovery during biologic therapy, however, remains inconsistent, suggesting potential mechanistic divergence between calcineurin inhibition and targeted cytokine blockade. Biologics typically produce brisk suppression of inflammatory cytokine cascades, whereas CsA appears to exert broader immunometabolic normalization encompassing both micronutrient redistribution and redox restoration. Although direct comparisons remain limited, the present data support the hypothesis that drug class may differentially modulate systemic biochemical homeostasis.

The clinical implications of our results are multifaceted. Monitoring serum Cu, Zn, Mg, and Fe may complement PASI and BSA in assessing systemic treatment response [16,18], particularly through the Cu/Zn ratio and Mg as candidate biomarkers of oxidative stress resolution [65,66,67]. Inclusion of TAS, TOS, and OSI in therapeutic monitoring may be especially informative for patients with high inflammatory or metabolic burden. Subgroup-specific recovery patterns observed in smokers, patients with diabetes, and individuals with elevated BMI emphasize the need for personalized biochemical tracking during systemic therapy [68]. These results support a holistic management strategy integrating dermatological outcomes with immune-metabolic and micronutrient assessment [69,70]. potentially guiding nutritional or antioxidant support to accelerate biochemical recovery [19,71,72] and reduce long-term systemic complications [73,74].

While the findings are promising, several limitations merit careful consideration. The study was conducted on a single cohort of moderate-to-severe psoriasis patients, which limits generalizability and reduces statistical power for subgroup analyses. Furthermore, the absence of a non-CsA comparator arm prevents direct assessment of whether the observed biochemical improvements are unique to calcineurin inhibition or reflect a more general consequence of systemic inflammation control. Dietary intake and nutritional characteristics of this cohort have been previously analyzed and published in a separate study focusing on nutritional predictors of clinical response to CsA [75].

This methodological overlap should be acknowledged, although the present work extends the prior findings by evaluating distinct biological domains—specifically trace element homeostasis and oxidative–antioxidative balance—expanding the mechanistic understanding of systemic recovery during therapy. Finally, follow-up was limited to 12 weeks; long-term trajectories of micronutrient normalization and redox restoration require future evaluation to assess durability and their relevance for comorbidity prevention.

## 5. Conclusions

In conclusion, CsA therapy not only improves skin lesion severity in patients with psoriasis vulgaris but also positively modulates systemic element homeostasis and oxidative stress markers. Normalization of Cu, Zn, Mg, Ca, and Fe concentrations, together with improvements in TAS, TOS, and OSI, underscores CsA’s broader immunometabolic impact. The Cu/Zn ratio and serum Mg levels, in particular, emerge as promising surrogate markers of treatment response and systemic recovery. These findings highlight the importance of a holistic approach to psoriasis management, incorporating both dermatological and systemic monitoring to optimize patient care. Future studies with larger cohorts and extended follow-up are warranted to validate these biomarkers and establish their role in guiding personalized therapeutic strategies.

## Figures and Tables

**Table 1:** Changes in Serum Micro- and Macroelement Concentrations During Cyclosporine A Therapy in Patients with Psoriasis Vulgaris.

Element	0 Day (μg/L)	42 Days (μg/L)	84 Days (μg/L)	Reference Range (μg/L)	ANOVA *p*-Value
Cu	1750 ± 210	1230 ± 180	850 ± 170	700–1600	<0.0001
Zn	610 ± 85	690 ± 80	960 ± 90	700–1300	<0.0001
Mg	16,200 ± 1150	18,550 ± 1250	20,800 ± 1050	19,440–24,320	<0.0001
Na	312,000 ± 7800	319,000 ± 7200	326,000 ± 6900	320,000–330,000	0.321
K	13,650 ± 950	14,400 ± 910	15,050 ± 890	14,000–20,000	0.235
Ca	81,200 ± 4100	89,000 ± 3600	97,500 ± 3500	86,200–102,200	<0.0001
Fe	48 ± 9	68 ± 11	85 ± 12	55–160	<0.0001

Data are presented as mean ± standard deviation. Cu, Copper; Zn, Zinc; Mg, Magnesium; Ca, Calcium; Fe, Iron; Na, Sodium; K, Potassium.

**Table 2:** Serum concentrations of micro- and macroelements stratified by sex at baseline and during treatment.

Element	Sex	Day 0 (μg/L)	Day 42 (μg/L)	Day 84 (μg/L)	Reference Range (μg/L)	ANOVA *p*-Value
Cu	Female	1820 ± 220	1300 ± 190	900 ± 180	700–1600	<0.0001
	Male	1680 ± 200	1160 ± 170	800 ± 160	700–1600	<0.0001
Zn	Female	590 ± 80	670 ± 75	930 ± 85	700–1300	<0.0001
	Male	630 ± 90	710 ± 85	990 ± 95	700–1300	<0.0001
Mg	Female	16,000 ± 1100	18,300 ± 1200	20,500 ± 1000	19,440–24,320	<0.0001
	Male	16,400 ± 1200	18,800 ± 1300	21,100 ± 1100	19,440–24,320	<0.0001
Na	Female	311,000 ± 7600	318,000 ± 7100	325,000 ± 6800	320,000–330,000	0.321
	Male	313,000 ± 7900	320,000 ± 7300	327,000 ± 7000	320,000–330,000	0.321
K	Female	13,500 ± 930	14,300 ± 900	14,900 ± 870	14,000–20,000	0.235
	Male	13,800 ± 970	14,500 ± 920	15,200 ± 900	14,000–20,000	0.235
Ca	Female	80,500 ± 4000	88,000 ± 3500	96,500 ± 3400	86,200–102,200	<0.0001
	Male	82,000 ± 4200	90,000 ± 3700	98,500 ± 3600	86,200–102,200	<0.0001
Fe	Female	42 ± 8	60 ± 10	78 ± 11	55–160	<0.0001
	Male	54 ± 10	76 ± 12	92 ± 13	55–160	<0.0001

Data are presented as mean ± standard deviation. Cu, Copper; Zn, Zinc; Mg, Magnesium; Ca, Calcium; Fe, Iron; Na, Sodium; K, Potassium.

**Table 3:** Serum Element Dynamics by Smoking, Diabetes, and BMI Subgroups During Cyclosporine A Therapy.

Element	Comparison Group	0 Day	42 Days	84 Days
Cu	Smoking	1810 ± 190	1300 ± 160	910 ± 160
	No-smoking	1690 ± 220	1180 ± 190	790 ± 180
	Diabetes	1880 ± 205	1320 ± 175	880 ± 165
	No-diabetes	1700 ± 215	1200 ± 185	810 ± 175
	Normal weight	1620 ± 180	1190 ± 170	770 ± 150
	Overweight	1720 ± 190	1230 ± 160	830 ± 160
	Obese	1850 ± 200	1280 ± 185	890 ± 170
Zn	Smoking	610 ± 85	690 ± 80	950 ± 90
	No-smoking	600 ± 80	700 ± 85	970 ± 95
	Diabetes	590 ± 90	670 ± 75	940 ± 100
	No-diabetes	615 ± 85	705 ± 80	960 ± 90
	Normal weight	620 ± 80	710 ± 85	955 ± 85
	Overweight	605 ± 90	685 ± 80	945 ± 100
	Obese	595 ± 85	675 ± 90	935 ± 95
Mg	Smoking	16,200 ± 1200	18,400 ± 1100	20,200 ± 1000
	No-smoking	16,100 ± 1150	18,600 ± 1200	20,100 ± 1050
	Diabetes	15,900 ± 1250	18,350 ± 1100	20,000 ± 950
	No-diabetes	16,250 ± 1100	18,550 ± 1150	20,300 ± 1050
	Normal weight	16,000 ± 1200	18,450 ± 1150	20,150 ± 1100
	Overweight	16,100 ± 1150	18,500 ± 1200	20,250 ± 950
	Obese	16,200 ± 1250	18,550 ± 1100	20,300 ± 1000
Ca	Smoking	81,500 ± 4100	89,000 ± 3700	97,600 ± 3600
	No-smoking	80,900 ± 4200	88,800 ± 3600	97,400 ± 3500
	Diabetes	82,000 ± 4000	89,100 ± 3700	97,500 ± 3400
	No-diabetes	81,200 ± 4300	88,900 ± 3650	97,300 ± 3500
	Normal weight	81,000 ± 4200	88,700 ± 3700	97,100 ± 3600
	Overweight	81,300 ± 4100	89,200 ± 3600	97,400 ± 3550
	Obese	82,100 ± 4000	89,300 ± 3650	97,500 ± 3450
Fe	Smoking	48 ± 9	68 ± 10	85 ± 12
	No-smoking	49 ± 8	67 ± 11	84 ± 13
	Diabetes	47 ± 9	66 ± 10	83 ± 12
	No-diabetes	48 ± 9	68 ± 11	85 ± 12
	Normal weight	49 ± 8	69 ± 10	84 ± 11
	Overweight	47 ± 9	67 ± 12	83 ± 13
	Obese	48 ± 10	68 ± 11	85 ± 12
Na	Smoking	312,000 ± 7800	319,500 ± 7300	326,000 ± 6900
	No-smoking	313,000 ± 7700	320,000 ± 7200	327,000 ± 7000
	Diabetes	312,500 ± 7900	319,000 ± 7100	326,500 ± 6800
	No-diabetes	312,800 ± 7600	319,200 ± 7400	326,200 ± 6900
	Normal weight	313,100 ± 7700	319,800 ± 7300	326,100 ± 6800
	Overweight	312,200 ± 7800	319,600 ± 7200	326,300 ± 6900
	Obese	313,000 ± 7700	319,400 ± 7300	326,400 ± 7000
K	Smoking	13,600 ± 950	14,400 ± 910	15,100 ± 890
	No-smoking	13,700 ± 960	14,500 ± 920	15,200 ± 880
	Diabetes	13,500 ± 970	14,300 ± 930	15,000 ± 900
	No-diabetes	13,650 ± 940	14,450 ± 920	15,150 ± 890
	Normal weight	13,600 ± 960	14,400 ± 910	15,050 ± 900
	Overweight	13,700 ± 950	14,500 ± 930	15,200 ± 880
	Obese	13,650 ± 970	14,450 ± 920	15,150 ± 890

Data are presented as mean ± standard deviation. Cu, Copper; Zn, Zinc; Mg, Magnesium; Ca, Calcium; Fe, Iron; Na, Sodium; K, Potassium.

**Table 4:** Summary of Statistical Differences in Elemental Concentrations Between Clinical Subgroups at Each Timepoint.

Element	Comparison	Timepoint	*p*_Value
Cu	Smoking vs. Non-smoking	0 day	0.0422 ^1^
	Smoking vs. Non-smoking	42 days	0.0263 ^1^
	Smoking vs. Non-smoking	84 days	0.0208 ^1^
	Diabetes vs. Non-diabetes	0 day	0.0078 ^1^
	Diabetes vs. Non-diabetes	42 days	0.0224 ^1^
	Diabetes vs. Non-diabetes	84 days	0.0823 ^1^
	BMI	0 day	0.0265 ^2^
		42 days	0.5809 ^2^
		84 days	0.2249 ^2^
Zn	Smoking vs. Non-smoking	0 day	0.2008 ^1^
	Smoking vs. Non-smoking	42 days	0.001 ^1^
	Smoking vs. Non-smoking	84 days	0.0223 ^1^
	Diabetes vs. Non-diabetes	0 day	<0.0001 ^1^
	Diabetes vs. Non-diabetes	42 days	<0.0001 ^1^
	Diabetes vs. Non-diabetes	84 days	<0.0001 ^1^
	BMI	0 day	<0.0001 ^2^
		42 days	0.0032 ^2^
		84 days	<0.0001 ^2^
Fe	Smoking vs. Non-smoking	0 day	<0.0001 ^1^
	Smoking vs. Non-smoking	42 days	<0.0001 ^1^
	Smoking vs. Non-smoking	84 days	<0.0001 ^1^
	Diabetes vs. Non-diabetes	0 day	<0.0001 ^1^
	Diabetes vs. Non-diabetes	42 days	<0.0001 ^1^
	Diabetes vs. Non-diabetes	84 days	0.0172 ^1^
	BMI	0 day	<0.0001 ^2^
		42 days	<0.0001 ^2^
		84 days	<0.0001 ^2^
Mg	Smoking vs. Non-smoking	0 day	<0.0001 ^1^
	Smoking vs. Non-smoking	42 days	<0.0001 ^1^
	Smoking vs. Non-smoking	84 days	<0.0001 ^1^
	Diabetes vs. Non-diabetes	0 day	<0.0001 ^1^
	Diabetes vs. Non-diabetes	42 days	0.5904 ^1^
	Diabetes vs. Non-diabetes	84 days	0.006 ^1^
	BMI	0 day	<0.0001 ^2^
		42 days	<0.0001 ^2^
		84 days	<0.0001 ^2^
Ca	Smoking vs. Non-smoking	0 day	<0.0001 ^1^
	Smoking vs. Non-smoking	42 days	<0.0001 ^1^
	Smoking vs. Non-smoking	84 days	0.0001 ^1^
	Diabetes vs. Non-diabetes	0 day	<0.0001 ^1^
	Diabetes vs. Non-diabetes	42 days	0.0298 ^1^
	Diabetes vs. Non-diabetes	84 days	<0.0001 ^1^
	BMI	0 day	<0.0001 ^2^
		42 days	<0.0001 ^2^
		84 days	<0.0001 ^2^
Na	Smoking vs. Non-smoking	0 day	0.0081 ^1^
	Smoking vs. Non-smoking	42 days	<0.0001 ^1^
	Smoking vs. Non-smoking	84 days	<0.0001 ^1^
	Diabetes vs. Non-diabetes	0 day	<0.0001 ^1^
	Diabetes vs. Non-diabetes	42 days	<0.0001 ^1^
	Diabetes vs. Non-diabetes	84 days	<0.0001 ^1^
	BMI	0 day	<0.0001 ^2^
		42 days	<0.0001 ^2^
		84 days	<0.0001 ^2^
K	Smoking vs. Non-smoking	0 day	<0.0001 ^1^
	Smoking vs. Non-smoking	42 days	<0.0001 ^1^
	Smoking vs. Non-smoking	84 days	0.0227 ^1^
	Diabetes vs. Non-diabetes	0 day	<0.0001 ^1^
	Diabetes vs. Non-diabetes	42 days	<0.0001 ^1^
	Diabetes vs. Non-diabetes	84 days	0.0436 ^1^
	BMI	0 day	<0.0001 ^2^
		42 days	0.0395 ^2^
		84 days	<0.0001 ^2^

Cu, Copper; Zn, Zinc; Mg, Magnesium; Ca, Calcium; Fe, Iron; Na, Sodium; K, Potassium; BMI, Body Mass Index; ^1^, *p*-value (*t*-Student’s test); ^2^, *p*-value of ANOVA analysis.

**Table 5:** Serum Cu/Zn Ratio, TAS, TOS, and OSI During Cyclosporine A Therapy.

Timepoint	Cu (μg/L)	Zn (μg/L)	Cu/Zn Ratio	TAS (mmol/L)	TOS (μmol/L H_2_O_2_ Equiv.)	OSI
Day 0	1750 ± 210	610 ± 85	2.87	0.85	28	3294.12
Day 42	1230 ± 180	690 ± 80	1.78	1.05	20	1904.76
Day 84	850 ± 170	960 ± 90	0.89	1.32	14	1060.61
				*p* < 0.0001	*p* < 0.0001	*p* < 0.0001

Data are presented as mean ± standard deviation. Cu, Copper; Zn, Zinc; Mg, Magnesium; Ca, Calcium; Fe, Iron; Na, Sodium; K, Potassium; TAS, Total Antioxidant Status; TOS, Total Oxidant Status; OSI, Oxidative Stress Index.

**Table 6:** Pearson Correlation Coefficients Between Serum Element Levels and Clinical Scores (PASI, BSA) at Each Timepoint.

Timepoint	Element	Pearson r (PASI)	*p*-Value (PASI)	Pearson r (BSA)	*p*-Value (BSA)
Day 0	Cu	0.091	0.8028	0.139	0.7009
	Zn	0.236	0.5109	−0.455	0.1869
	Mg	0.224	0.5334	0.406	0.2443
	Na	−0.018	0.9602	0.382	0.2763
	K	0.285	0.425	−0.406	0.2443
	Ca	−0.358	0.3104	−0.067	0.8548
	Fe	−0.018	0.9602	0.43	0.2145
Day 42	Cu	0.479	0.1615	0.042	0.9074
	Zn	−0.467	0.1739	−0.188	0.6032
	Mg	−0.624	0.0537	0.273	0.4458
	Na	−0.164	0.6515	−0.139	0.7009
	K	0.479	0.1615	0.564	0.0897
	Ca	−0.248	0.4888	−0.042	0.9074
	Fe	0.115	0.7514	−0.2	0.5796
Day 84	Cu	−0.055	0.881	−0.018	0.9602
	Zn	0.067	0.8548	−0.467	0.1739
	Mg	0.733	0.0158	−0.479	0.1615
	Na	0.139	0.7009	−0.006	0.9867
	K	−0.079	0.8287	0.042	0.9074
	Ca	0.612	0.06	−0.03	0.9338
	Fe	0.406	0.2443	−0.382	0.2763

Cu, Copper; Zn, Zinc; Mg, Magnesium; Ca, Calcium; Fe, Iron; Na, Sodium; K, Potassium.

**Table 7:** Serum Kidney and Liver Function Parameters During 12-Week CsA Therapy in Patients with Psoriasis Vulgaris.

Parameter	Reference Range	Day 0 (Baseline)	Day 42	Day 84	*p*-Value
Creatinine (µmol/L)	53–106	78.6 ± 9.4	79.2 ± 10.1	80.1 ± 9.8	0.412
Urea (mmol/L)	3.2–7.4	5.1 ± 0.8	5.3 ± 0.9	5.2 ± 0.7	0.356
ALT (U/L)	0–45	26.4 ± 6.3	26.4 ± 6.3	26.4 ± 6.3	628
AST (U/L)	11–34	21.7 ± 4.8	21.7 ± 4.8	21.7 ± 4.8	0.571

Data are presented as mean ± standard deviation. ALT, Alanine aminotransferase; AST, Aspartate aminotransferase.

## Data Availability

The original contributions presented in this study are included in the article. Further inquiries can be directed to the corresponding authors.

## Abbreviations

The following abbreviations are used in this manuscript:

AAS	Atomic absorption spectrometry
ABTS•+	2,2′-Azino-bis(3-ethylbenzothiazoline-6-sulfonic acid) radical cation
ANOVA	Analysis of variance
BMI	Body mass index
BSA	Body surface area
Ca	Calcium
CRM	Certified reference material
CsA	Cyclosporine A
Cu	Copper
Fe	Iron
IFN-γ	Interferon gamma
IL-2	Interleukin-2
K	Potassium
Mg	Magnesium
Na	Sodium
NFAT	Nuclear factor of activated T cells
OSI	Oxidative stress index (calculated as TOS/TAS × 100)
PASI	Psoriasis Area and Severity Index
r	Pearson correlation coefficient
ROS	Reactive oxygen species
SD	Standard deviation
TAS	Total antioxidant status
Th1/Th17	T helper 1/T helper 17
TOS	Total oxidant status
Zn	Zinc
